# Modulation of Glia-Mediated Processes by Spinal Cord Stimulation in Animal Models of Neuropathic Pain

**DOI:** 10.3389/fpain.2021.702906

**Published:** 2021-07-14

**Authors:** David L. Cedeño, Courtney A. Kelley, Krishnan Chakravarthy, Ricardo Vallejo

**Affiliations:** ^1^Research and Development, Lumbrera LLC, Bloomington, IL, United States; ^2^Department of Psychology, Illinois Wesleyan University, Bloomington, IL, United States; ^3^Deparment of Anesthesiology and Pain Medicine, University of California, San Diego, La Jolla, CA, United States; ^4^Research Department, National Spine and Pain Center, Bloomington, IL, United States

**Keywords:** spinal cord stimulation, neuropathic pain, animal models, mechanism of action, neuronal-glial interactions

## Abstract

Glial cells play an essential role in maintaining the proper functioning of the nervous system. They are more abundant than neurons in most neural tissues and provide metabolic and catabolic regulation, maintaining the homeostatic balance at the synapse. Chronic pain is generated and sustained by the disruption of glia-mediated processes in the central nervous system resulting in unbalanced neuron–glial interactions. Animal models of neuropathic pain have been used to demonstrate that changes in immune and neuroinflammatory processes occur in the course of pain chronification. Spinal cord stimulation (SCS) is an electrical neuromodulation therapy proven safe and effective for treating intractable chronic pain. Traditional SCS therapies were developed based on the gate control theory of pain and rely on stimulating large Aβ neurons to induce paresthesia in the painful dermatome intended to mask nociceptive input carried out by small sensory neurons. A paradigm shift was introduced with SCS treatments that do not require paresthesia to provide effective pain relief. Efforts to understand the mechanism of action of SCS have considered the role of glial cells and the effect of electrical parameters on neuron–glial interactions. Recent work has provided evidence that SCS affects expression levels of glia-related genes and proteins. This inspired the development of a differential target multiplexed programming (DTMP) approach using electrical signals that can rebalance neuroglial interactions by targeting neurons and glial cells differentially. Our group pioneered the utilization of transcriptomic and proteomic analyses to identify the mechanism of action by which SCS works, emphasizing the DTMP approach. This is an account of evidence demonstrating the effect of SCS on glia-mediated processes using neuropathic pain models, emphasizing studies that rely on the evaluation of large sets of genes and proteins. We show that SCS using a DTMP approach strongly affects the expression of neuron and glia-specific transcriptomes while modulating them toward expression levels of healthy animals. The ability of DTMP to modulate key genes and proteins involved in glia-mediated processes affected by pain toward levels found in uninjured animals demonstrates a shift in the neuron–glial environment promoting analgesia.

## Introduction

Pain is a natural reflex that protects an individual from potentially harmful stimuli. Specialized nerve terminals conduct information from the periphery and internal organs to the brain *via* sensory ganglia and distinct tracts in the spinal cord (SC). When a certain stimulus (mechanical, chemical, thermal, emotional) exceeds a particular threshold, the brain interprets it as pain. Pain is a complex quale encompassing a concerted and balanced interplay of biological processes orchestrated through cellular signaling and interactions throughout the entire nervous system. Acute pain accompanies injuries and is necessary to initiate and sustain a healing and self-protection process to return the damaged tissues to normality. Once the affected part of the body is healed, pain recedes, and the system goes back into balance. However, many individuals continue to experience pain beyond what constitutes the normal healing process from injuries. In this case, persistent or chronic pain sets in due to distorted and unbalanced processing of events. Our understanding of chronic or persistent pain, although limited, has evolved greatly in the last 60 years. Many pain theories have been developed (1, 2), most of them centered on neuronal processing, driven by the fact that neurons are the main carriers of sensory information to the brain. Indeed, the most recent of these, gate control theory (GCT) (3), has served as the foundational development of electrical neuromodulation therapies such as spinal cord stimulation (SCS), dorsal root ganglion stimulation (DRGS), and peripheral nerve stimulation (PNS) for the treatment of intractable chronic neuropathic pain (4–6). Conventional modalities of these treatments consist of applying electric signals to the dorsal columns of the SC, or the DRG, or a peripheral nerve to induce paresthesias that are steered to overlap the affected pain dermatomes. These paresthesias result from the stimulation of neurons in Aβ fibers and are intended to gate out the noxious input transmitted through small, unmyelinated, and slow conductive fibers (7). It is also plausible that conventional SCS exerts an inhibitory effect on wide dynamic range neurons *via* Aβ fibers or directly on these fibers, which are also known to contribute to neuropathic pain (8, 9). Caylor et al. provide a comprehensive review of the various mechanisms of action of SCS (10).

SCS has been proven to be an effective and safe reversible treatment of intractable chronic neuropathic pain of the trunk and limbs (11). Rooted in the foundations of the GCT of pain, it was developed to target neurons to induce paresthesia in the painful area, with electrical signals pulsed at a rate of 40-60 Hz. Technological developments of this paresthesia-based traditional SCS modality have resulted in improvements in clinical outcomes in which ~50% of treated patients with post-laminectomy pain syndrome obtained ≥50% pain relief (12). Other developments based on the utilization of electrical signals that do not rely on paresthesia have flourished in the last decade (13). Electrical pulses delivered at rates above what was traditionally utilized served as the foundational basis for the development of therapies that use bursts (pulses at 500 Hz delivered every 25 μs) or faster-uninterrupted pulses (>1 kHz). One of such treatments that utilized 10 kHz pulses provided superior outcomes (~80% of patients obtained ≥50% pain relief) relative to treatment with traditional SCS (14). These results spurred the review of the mechanism of actions that had remained centered on the neuronal doctrine embedded in further developments of the GCT (15).

A largely ignored fact in electrical neuromodulation is the now well-established key role of glial cells in the pathology of chronic pain (16, 17). In a pain state, microglia, the resident immune cells of the central nervous system (CNS), become activated into various phenotypes that promote pro-inflammatory and anti-inflammatory processes *via* the expression and release of cytokines, chemokines, and gliotransmitters (18). Intracellular activation of signaling cascades may cause these changes to become persistent by perpetuating an inflammatory state. Activation of inflammatory processes also triggers the activation of astrocytes, the glial components of the tripartite synapse. These cells modulate calcium signaling, regulate extracellular potassium, and buffer the effect of neurotransmitters (19). Astrocytes monitor the homeostasis of the synaptic clefts and provide neuronal nutrients (glutamine and L-serine) used in the synthesis of neurotransmitters (glutamate, GABA, glycine, and D-serine). Astrocytes communicate *via* calcium waves through gap junctions and regulate calcium-mediated processes central to the signaling of immune and inflammatory processes. They also capture nutrients from the circulatory system and regulate blood supply at the blood–brain barrier while releasing vasoactive molecules. These cells are associated with maintaining a chronic pain state as key players in the long-term potentiation of nociception. In addition to microglia and astrocytes, oligodendrocytes are now recognized to be involved in chronic pain (20, 21). Mature oligodendrocytes myelinate neuronal axons and thus play an important role in maintaining proper signal conduction. Precursor oligodendrocyte cells (OPCs) are mobile and are known to populate and migrate from white and gray matter. These cells can mature to become myelinating as required by the CNS and assist astrocytes via cell-to-cell signaling processes in monitoring the homeostatic balance of the system.

Over a decade ago, Vallejo et al. (22) suggested that electrical stimulation of neural tissue could also target glial cells, acknowledging that they play a fundamental role in the establishment and maintenance of neuropathic pain. This idea has been supported by reports demonstrating that astrocytes and oligodendrocytes respond to electrical stimulation and that such response may be modulated by modifying the characteristics of the electric signal (23–26). Furthermore, Sluka et al. (27) showed that standard low rate (LR) SCS reversed the expression of protein markers associated with glial activation in a rat spared nerve injury (SNI) model of neuropathic pain. Vallejo et al. (28) later showed, using high-throughput transcriptomics, that LR SCS affected the expression of hundreds of genes associated with neuroinflammation, immune response, and ion transport regulation, among others, in the SNI model. Similar results were obtained by Guan et al. (29, 30) using transcriptomic-based analysis of the effects of LR SCS in the rat chronic constricted injury (CCI) model and the rat chemotherapy-induced painful neuropathy (CIPN) model. These studies also validated the involvement of glial cells in immune response and inflammatory processes associated with the pain models. Vallejo et al. (31–33) have used the knowledge obtained from their molecular biology-based research to develop an SCS approach in which multiple signals are multiplexed to target neurons and glial cells differentially. This approach has been successfully translated to the clinic (34), in which differential target multiplexed SCS programs have provided superior pain relief (~80% of subjects with ≥50% relief) relative to traditional SCS.

This manuscript provides insight into what has been learned using animal models of SCS on the modulation of glial-based processes, emphasizing evidence obtained using molecular biology methods.

## Materials and Methods

### Animal Models in Spinal Cord Stimulation Used for Glial-Mediated Processes

Animal models for SCS have been recently reviewed (35). Three pain models have mostly been used for studying the molecular effects of SCS on neuropathic pain.

#### SCS in the Spared Nerve Injury Model

Details of the implementation of the SNI model for SCS are provided by Vallejo et al. (31, 36). The model targets the sciatic nerve at the point of trifurcation into the peroneal, tibial, and sural nerves in the hindlimb of the animal, located under the biceps femoris muscle. Both the tibial and peroneal nerves were ligated with a silk suture, and 2–4 mm of the nerve was sectioned and removed, leaving the sural nerve intact. Nerve injury caused long-lasting mechanical and thermal hypersensitization in the operated limb. For SCS, a cylindrical quadripolar lead was implanted in the dorsal epidural space of the L1-L2 vertebral level *via* a laminectomy at the L4 level. The lead cable was securely anchored to the muscle tissue around the L5 spinal process to reduce migration risk. The lead cable terminals were connected to a block with an ethernet plug attached to a custom-made harness. An ethernet spiral cable connected the block to an assembly that was connected to an external neurostimulator, which delivered the electrical signals to be studied. This setup is capable of providing continuous SCS for many days. In their work, Vallejo et al. (31) have studied various SCS modalities, including traditional low rate (LR, 50 Hz, 20 or 150 μs pulse width, PW), high rate (HR, 1.2 kHz, 50 μs PW), or differential target multiplexed programming (DTMP, 50 Hz and 1.2 kHz, 50 and 150 μs PW). Current intensities were set at 70% of the motor threshold (MT). The effects were studied at an early stage of the pain model, as SCS was started 5 days post-SNI surgery.

The SNI was also similarly implemented by Sluka et al., except for using a quadripolar paddle lead implanted *via* laminectomy at the T13 level (27, 37). Signals were pulsed at 4 or 60 Hz with voltage intensities set at 90% of the MT and delivered 6 h a day for 4 days. PW was likely 250 μs based on another report from this group that utilized the same rate and intensity (37). SCS was started 2 weeks post-nerve injury.

#### SCS in the Chronic Constriction Injury Model

Details of the implementation of the CCI for SCS are provided by Guan et al. (29, 38). The model targets the sciatic nerve in the hindlimb of the animal located under the biceps femoris muscle. Rather than axotomizing the nerve branches, the sciatic nerve trunk proximal to the trifurcation is loosely ligated with four 4-0 silk sutures about 0.5 mm apart. Nerve injury also developed into a stable and persistent pain model. A quadripolar paddle lead covering the T13-L1 vertebral levels was epidurally implanted *via* a laminectomy at the T13 level in this implementation. Lead cables were tunneled subcutaneously rostrally to exit at the cervical level near the head. These were connected to an external neurostimulator which delivered electrical signals at 50 Hz, 200 μs PW, and current intensities set to 80% of the MT. SCS was delivered twice a day (2 h per session) for 3.5 consecutive days. SCS was started 36 days post-nerve injury.

#### SCS in the Chemotherapy-Induced Painful Neuropathy Model

Details of the implementation of the CIPN for SCS are provided by Guan et al. (30). This model uses intraperitoneal administration of paclitaxel (1.5 mg/kg) for 4 consecutive days. Animals reached maximum hypersensitivity manifested in the limbs about 2 days after the final dose of paclitaxel. A quadripolar paddle lead was epidurally implanted via a laminectomy at the T13 vertebral level. The lead-covered the dorsal T13-L1 levels. Lead cables were tunneled subcutaneously rostrally to exit near the head. These were connected to an external neurostimulator which delivered electrical signals at 50 Hz, 200 μs PW, and current intensities set to 80% of the MT. SCS was delivered preemptively for 14 days (6–8 h per day), starting 1 day before starting paclitaxel injection.

### Molecular Biology Methods

#### Transcriptomics Using Microarrays

High-throughput quantification of gene expression using microarray technology was used by Vallejo et al. (28) to study the effects of traditional LR SCS (see section SCS in the Spared Nerve Injury Model above) on the stimulated SC section (dorsal ipsilateral quadrant) and the L5 DRG of SNI animals and uninjured animals. This was the first time the transcriptome of SCS was reported. RNA from 48 samples (2-5 from 6 experimental groups) was extracted and quantitated from frozen tissue, preserved in RNAlater solution, using standard procedures (39). The RNA was hybridized to Agilent rat gene expression 4 × 44 microarray kits. Half of the samples were labeled with Cy5 and the other half with Cy3 fluorescent dyes. When a particular hybridized RNA of the sample is complementary to the cDNA probe in the microarray, the fluorescent dye is activated and detected using optical methods. The arrays used in this work interrogated 26,930 genes using 30,367 probes in the microarrays.

#### Transcriptomics Using RNA Sequencing

Although microarray analysis is a convenient way of quantifying a large amount of protein-coding messenger RNAs (mRNAs), it is limited to those genes that have been characterized and built into the microarrays. In contrast, RNA sequencing allows sampling of the total RNA in a sample, including mRNA, micro RNAs (miRNAs), and long non-coding RNAs (lncRNAs). This methodology was used first by Guan et al. (29) to determine the effect of traditional LR SCS on the CCI (see section SCS in the Chronic Constriction Injury Model above) and later by the same group (30) to study the effect of LR SCS on CIPN (see section SCS in the Chemotherapy-Induced Painful Neuropathy Model above). Ipsilateral L4–L6 SC segments were dissected and stored in a DNA/RNA shield solution. RNA was extracted and quantitated using standard methods. Five hundred ng of total RNA was used to build strand-specific sequencing libraries. These were built after polyadenyl [poly(A)] selection of mRNA using a commercial kit. Samples were barcoded using a kit that contains adapters and primers designed for high amplification efficiency. The RNA sequencing libraries were quantified using quantitative polymerase chain reaction (qPCR). Libraries were normalized, pooled, and sequenced in an Illumina HiSeq4000 to a depth of 33.6 million reads per sample.

Recently, Vallejo et al. (31) used RNA sequencing to study the effect of DTMP on gene expression in the stimulated ipsilateral cord compared with LR and HR SCS in SNI animals (see section SCS in the Spared Nerve Injury Model above). RNA from frozen dorsal ipsilateral quadrants of the L1-L2 SCs of 20 animals (4 per experimental group) stored in RNAlater was isolated using the TriZol commercial kit. RNA libraries were constructed using a commercial kit after poly(A) enrichment of mRNA from a 1 μg sample of total RNA. RNA was coded by chemically fragmenting the mRNA, annealing with random hexamers, and converting to double-stranded cDNA ligated to indexed adaptors. The cDNA was amplified, quantitated, and polled using qPCR. Pooled barcoded libraries were sequenced in an Illumina HiSeq 4000 and quality controlled using software algorithms that select and map mRNAs to the rat genome (NCBI Rnor_6.0 Annotation Release 106).

#### Gene Expression Using Reverse Transcription qPCR

Vallejo et al. (40) used quantitative reverse transcription PCR (RT-qPCR) to study the modulatory effects of phase polarity and extent of anodic charge of LR signals (50 Hz, 50 μs cathodic PW, the intensity at 66% of the MT) on a panel of 21 genes associated with glial-related processes. Vallejo et al. (28) and Guan et al. (29) also used RT-qPCR to validate their high throughput results. Transcripts from the genes of interest were identified in the rat genome to design sequence-specific primers using bioinformatic tools (41). Column-purified RNA extracted from experimental samples was reverse-transcribed into first-strand cDNA using a commercial kit. Quantitation was carried out by amplification of cDNA using qPCR. A mix of cDNA, reverse and forward primers, polymerase, deoxy-nucleotide triphosphates (dNTPs), and a fluorescent DNA-intercalation probe was thermally cycled under appropriate conditions. Gene expression levels were quantitated in triplicate by measuring the C_q_ values from the thermal amplification cycles using the ΔΔC_q_ method (42). An internal control gene was used to normalize the expression of genes of interest, obtaining a ΔC_q_. Differential gene expression between experimental groups compared their corresponding ΔC_q_ values and obtaining a ΔΔC_q_.

#### Immunohistochemistry

Immunohistochemistry was used by Sluka et al. (27) as well as Guan et al. (38) to detect proteins associated with glial activation related to treatment with traditional LR SCS (see section SCS in the Spared Nerve Injury Model above). Anesthetized rats were transcardially perfused with heparinized saline (10%) followed by paraformaldehyde (4%) with 15% picric acid. SCs were dissected and fixed for 1 h in paraformaldehyde and frozen after immersion in 30% sucrose. Sections (20 μm thick) were frozen cut onto slides for staining. Before exposure to specific antibodies, slides were blocked with 3% goat serum followed by avidin-biotin. Overnight exposure to anti-mouse GFAP antibodies (1:5,000) and goat anti-Rabbit MCP-1 antibodies (1:500) were used to stain active astrocytes. GFAP and MCP-1 staining were developed by exposing slides to biotinylated goat anti-mouse IgG and goat and goat anti-rabbit IgG, respectively, followed by exposure to Streptavidin-488 for fluorescent detection. Active microglia were stained by exposure to anti-mouse OX-42 antibodies (1:2,500) and developed using the same process for developing astrocyte stains. Slides from five animals per group were imaged using fluorescence microscopy and analyzed using imaging software (ImageJ) to differentially quantify the amount of antibodies in stimulated and non-stimulated cords.

#### High-Throughput Proteomics and Phosphoproteomics

Vallejo et al. (43) pioneered high throughput protein profiling methods for studying the effect of traditional LR SCS on the SNI model of neuropathic pain. The global proteomic analysis used by this group identified and quantitated proteins in a sample using liquid chromatography and tandem mass spectrometry (LC/MS/MS). Proteins from SC tissues (see section SCS in the Spared Nerve Injury Model above) were separated, purified, and quantitated using standard procedures that used non-ionic buffers compatible with the LC method (44). Proteins were trypsinized after reduction and alkylation of cysteine residues. Tryptic peptides were purified and desalted, and labeled with isotopic tags (TMT 10plex) for simultaneous processing and quantitation. Labeled samples were equally mixed and separated using LC into 18 fractions. Each fraction was then subjected to LC/MS/MS in quadruplicate. Mass spectra of tagged peptides were searched against the Uniprot curated proteome of the rat to identify proteins based on unique peptide profiles using bioinformatics software (45). Identified proteins were quantified from normalized spectral intensities of their unique peptides.

Post-translational modifications of proteins serve as a diverse source of regulatory and signaling moieties. Protein phosphorylation by kinases is one such process. Phosphoproteomics has been used to investigate glia-mediated regulation of pain-related processes. Phosphorylated proteins are enriched from the total protein sample *via* reversed-phase solid-phase extraction, followed by phospho-enrichment using immobilized metal affinity chromatography (IMAC) (46) with iron-based magnetic beads (PTMScan® Fe-IMAC, Cell Signaling Technology, Danvers MA). Unbound peptides were washed out, and immobilized phosphopeptides eluted with basic pH buffer. Reversed-phase purification was performed before LC/MS/MS analysis, carried out as described above for whole proteomics.

### Bioinformatics and Statistical Analyses

Using high-throughput methods is the vast amount of data that needs to be analyzed to gain useful insight. Various algorithms have been created for mining the data using curated databases (47). Vallejo et al. (28, 31) have used weighted gene coexpression network analysis (WGCNA) (48) to catalog genes according to their expression trends based on the various treatment groups. The WGCNA groups genes in a hierarchical fashion in modules. Pairwise comparisons between expression patterns in a given module for treatment groups are based on an eigengene value, representing the degree of variance. Significance *P*-values for multiple comparisons of eigengenes were corrected using the false discovery rate (FDR) method (49).

To determine the biological relevance of genes in a module, individual modules were subjected to a gene ontology enrichment analysis (GOEA), emphasizing their involvement in biological processes curated into gene ontology terms. Various software options are used to carry out the GOEA (50). The method ranks the GO terms based on the number of genes in the module (i.e., experimental gene set) represented in a given curated GO term. Significance *P*-values for multiple comparisons based on the ranks were corrected using the FDR method.

Protein interaction network maps can be constructed using web-based bioinformatics tools, such as string-db (51), generating them from curated literature reports. Tools can also provide clustering based on connectivity indexing and GOEA and analysis based on Reactome and protein domains, which allow categorization of proteins based on their biological relevance. The FDR method is used to rank the results based on significance.

### Experimental Designs

Experimental designs were well-controlled, including at a minimum a stimulation sham (SCS turned off). Others included naïve and injury sham (uninjured) animals. Table 1 summarizes the experimental designs of the work cited in the previous sections.

**Table 1:** Summary of experimental designs in preclinical investigations of SCS.

**Model**	**Tissues analyzed**	**SCS treatments**	**Controls**	**SCS time**	**References.**
SNI (14d)	SC^*a*^	LR (4 Hz, 250 μs, 90% MT) LR (60 Hz, 250 μs, 90% MT) At T10-T12	No-SCS	6 h/day for 4 days	Sato et al. (27)
SNI (4d)	L1-L2 SC L5 DRG	LR (50 Hz, 20 μs PW, 70% MT) At L1-L2	No-SCS No-SNI (implanted) SNI (no implant) Sham for SNI (no implant)	72 h continuous	Vallejo et al. (28)
SNI (5d)	L1-L2 SC	DTMP (50 Hz/1.2 kHz, 50/150 μs PW, 70% MT) LR (50 Hz, 150 μs PW, 70% MT) HR (1.2 kHz, 50 μs, 70% MT) At L1-L2	No-SCS Naïve	48 h continuous	Vallejo et al. (31)
SNI (5d)	L1-L2 SC	LR (50 Hz, 50 μs PW cathodic, variable anodic PW, 66% MT) At L1-L2	No-SCS No-SNI	24 h continuous	Vallejo et al. (40)
CCI (36d)	L4-L6 SC	LR (50 Hz, 200 μs PW, 80% MT) At T10-T12	No-SCS	2 h/day for 3.5 days	Stephens (29)
CCI (18d)	L4-L6 SC	LR (50 Hz, 200 μs PW, 80% MT) At T10-T12	No-SCS	3 h per session (2 per day) for 3.5 days	Shu et al. (38)
CIPN	L4-L6 SC	LR (50 Hz, 200 μs PW, 80% MT) At T10-T12	No-SCS CIPN (no implant) Naïve	6-8 h/day for 14 days^*b*^	Sivanesan et al. (30)

a *Vertebral levels analyzed were not reported*.

b *Started preemptively 1 day before inducing the pain model*.

## Results and Discussion

### Modulation of Glia-Related Gene Expression by SCS

Transcriptomics and qPCR have been used to study the modulatory effects of SCS in SC and DRG tissues. In their transcriptomics work on the SNI, Vallejo et al. (28) showed that 72 h of continuous treatment with traditional LR SCS modulated gene expression in the SC related to neuroinflammation and immune response. This included glia-related genes such as *Lyz2, Cd68, Cd74, Cxcl16, RT1-Bb, RT1-Da, RT1-Db1, Tlr2, Itgb2, Aif1*, and *Tspo*. Some of these genes are markers of microglial activation (e.g., *Cd68, Cd74, Itgb2*) and astrocyte activation (e.g., *Tlr2, Cxcl16*), which are usually elevated by nerve injury and the inflammatory process. Interestingly, LR SCS elevated the expression of these genes. Furthermore, the increase occurs in the absence of injury for some of these genes, implying that LR SCS may activate glial cells. Transcriptomics work reported by Guan et al. (29) on the effect of LR SCS on the CCI are remarkably similar, despite the differences in pain models, their chronicity, stimulation times, and SC segments analyzed. This work found that traditional LR SCS upregulated immune- and inflammatory-related processes and that this treatment elevated expression of markers of astrocyte (*Gfap, Ccl2*) and microglia (*Cd68, Itgam*) activation in the SC, caudal to the stimulation site. This group followed up with one study (36) in which they measured microglia mRNA markers for specific M1-like (pro-inflammatory) and M2-like (anti-inflammatory) phenotypes in the L4–L6 SC. They found that expression levels of two M1-like markers (*Cd16* and *Cd32*) were significantly elevated by the CCI, while *Cd16* was further increased by low rate SCS. The expression levels of the M1-like marker *i-Nos* were elevated by treatment but were not by the CCI. None of the three M2-like markers (*Arg1, Cd163*, and *Tgfb*) were affected by either the CCI or SCS. These authors also showed that intrathecal administration of a low dose (.067 μg/μl) of the microglia inhibitor, minocycline, in conjunction with LR SCS, provided analgesic effect after acute (2 h) SCS. Cedeño et al. (32) analyzed a microglia-specific transcriptome (101 genes) and found that the SNI upregulated 79% of these genes relative to naïve animals and that LR SCS reversed the expression levels of only 23% of genes toward the naïve level, in contrast with what was obtained with DTMP and HR SCS. Indeed, they found that the expression profile of the microglia transcriptome associated with LR SCS treatment negatively correlated with the expression profile of naïve animals. This group followed up with a report (33) in which larger microglia transcriptomes associated with resting (1,569 genes), post-injury (3,706 genes), and neuroprotective (1,588 genes) states were analyzed. They found that expression levels relative to SNI in both post-injury and neuroprotective states upon LR SCS correlated weakly with those found for naïve animals. This was consistent with the fact that only ~50% of genes in these transcriptomes returned toward naïve levels upon treatment with LR SCS. Glial activation induces the release of pro-inflammatory cytokines (such as *Tnfa, Il1b*, and *Il6*) and a reduction of anti-inflammatory ones (such as *Il10* and *Il4*). Tilley et al. (52, 53) measured some of these in stimulated SC and the L5 DRG of SNI animals and those treated with LR SCS using RT-qPCR. They found that the SNI elevated the expression level of *Tnfa* in the SC relative to injury-sham. Furthermore, they found that LR SCS further increased *Tnfa* expression in both injured and uninjured animals. Interestingly, changes in *Tnfa* correlated well with the changes observed in the microglial activation marker, *Itgam*, and the astrocyte activation marker, *Gfap*. *Itgam* was also increased in the L5 DRG due to the SNI and further increased by LR SCS, while the level of *Il1b* was significantly elevated in the DRG due to LR SCS. Shu et al. (38) also found that LR SCS significantly increased *il1b* in the L4-L6 SC of CCI animals. However, *Tnfa* levels were not changed. It is noteworthy to highlight the similarity of the findings by Tilley et al. in the DRG and those by Shu et al. in the L4-L6 SC, considering that SCS was applied more rostrally. Thus, preclinical results based on transcriptomics suggest that traditional LR SCS may enhance microglial activation. Based on molecular biology, these reports complement the existing mechanisms of action of conventional SCS, which have mostly been centered on the effect of electrical stimulation on neuronal activity (10). The modulation of glial cell activity by SCS suggests that the mechanisms of action should also account for these cells in their interactions with neurons and their combined contribution to neuropathic pain.

**Figure 1:**
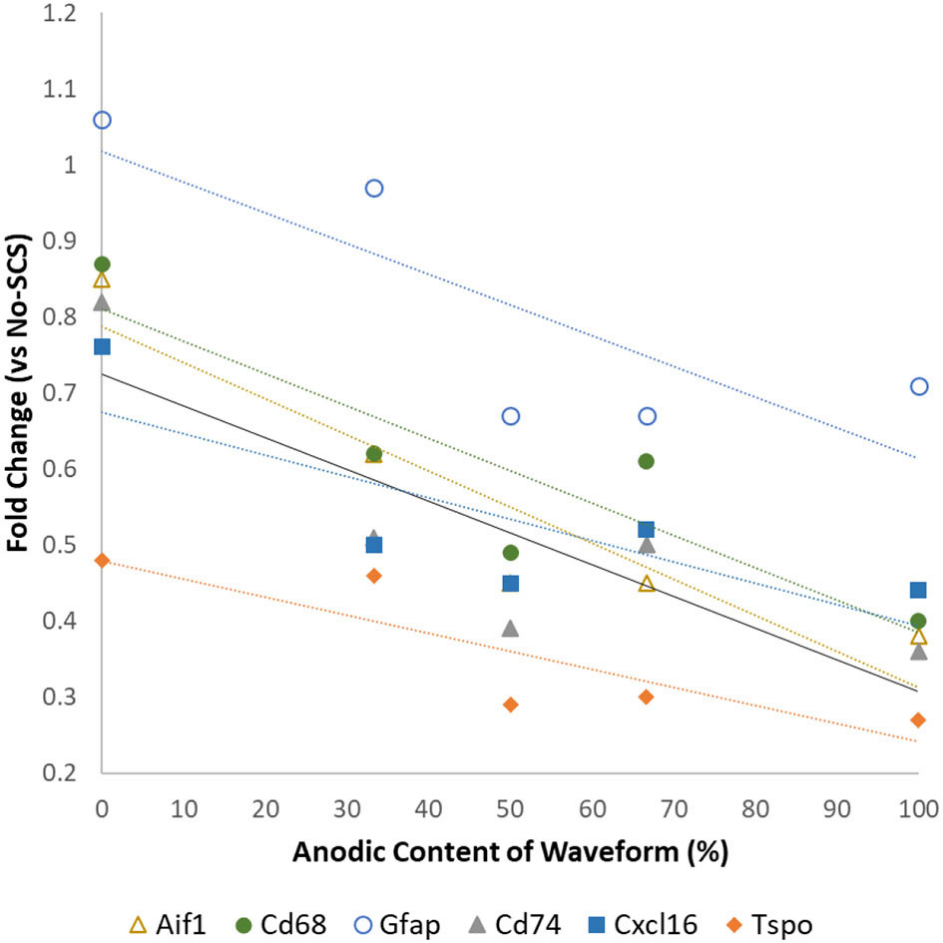
Differential expression levels of mRNA associated with glial activation as a function of the anodic content of pulsed signals at a low rate (50 Hz, 50 μs PW cathodic, 66% MT). Data from Vallejo et al. (40).

Vallejo et al. (40) found that the expression levels of glia-related genes could be modulated by modifying the anodic content of the electrical signal. Although this may not have a direct clinical application due to the charge unbalanced signals, it illustrates that the properties of electrical signals may influence glial activation and, thus, its effects on neuroinflammation and neuropathic pain relief. Figure 1 shows that an increase in the amount of anodic charge in a bipolar signal correlates strongly with a decrease in the expression levels of glial activation markers *Aif1, Gfap, Cd68, Tspo, Cd74*, and *Cxcl16*.

These findings are congruent with previous evidence demonstrating that glial cells respond to the application of electric fields. Roitbak and Fanardjian (54) showed that the membrane of cortex astrocytes of a cat could be depolarized by changing the parameters (intensity, polarity, and rate) of pulsed electrical signals. Lee et al. (23–25) also demonstrated that electrical stimulation of astrocytes in the brain of rodents induced the release of glutamate. This release is dependent on the properties of the electric signal, including rate, pulse width, intensity, and extent of charge balance. Yamazaki et al. (55) also demonstrated that electrical stimulation of oligodendrocytes could modulate conduction velocities in the axons they myelinate. Another important fact is that glial cells are the most abundant cells in the spinal cord (56, 57). A recent human anatomical study showed about 13 glial cells per every neuronal soma in the gray matter of the dorsal horn closest to the SCS field in the T8-T11 levels (57).

**Figure 2:**
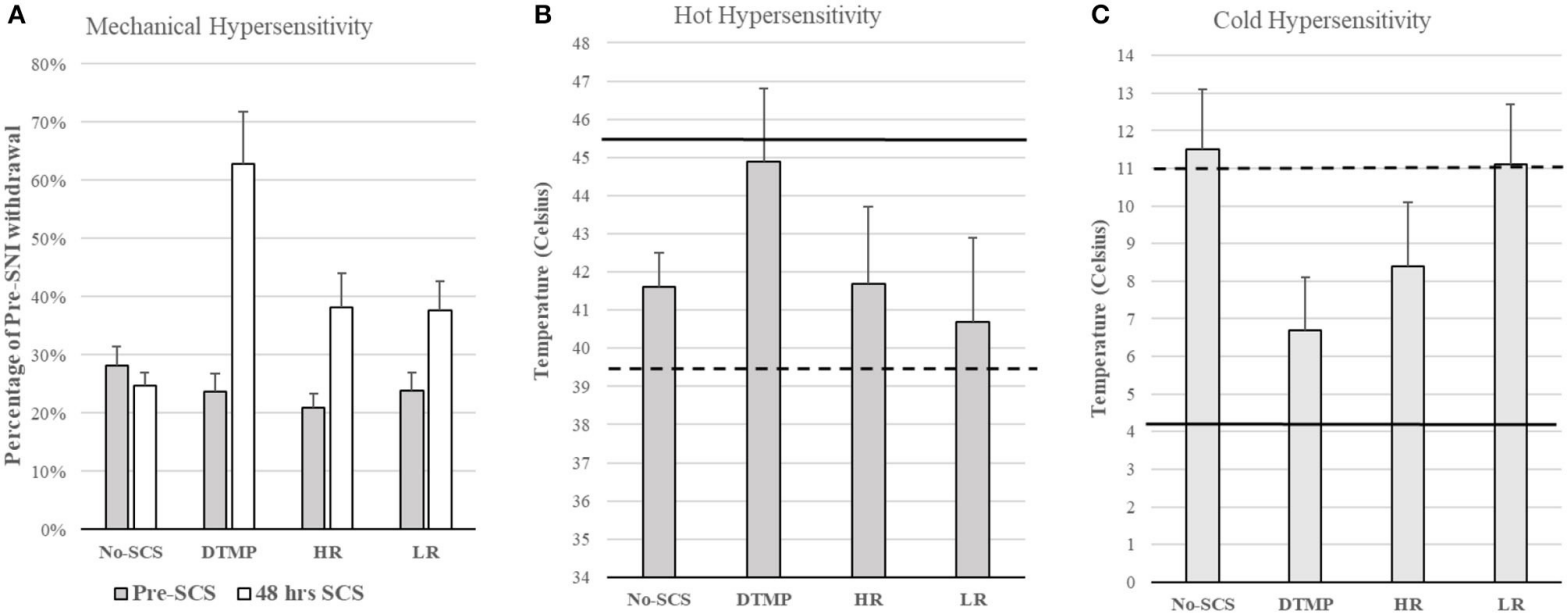
**(A)** Mechanical hypersensitivity relative to Pre-SNI. **(B)** Hot hypersensitivity. The dashed line denotes the mean of all animals pre-SCS, Continuous line denotes the mean baseline of all animals (pre-SNI). **(C)** Cold hypersensitivity. The dashed line denotes the mean of all animals pre-SCS, Continuous line denotes the mean baseline of all animals (pre-SNI). Figures reproduced from Vallejo et al. (31). SNI, spared nerve injury; SCS, spinal cord stimulation.

Considering that glial cells are the most abundant in the spinal cord, play a fundamental role in chronic pain, and are electrically excitable, Vallejo et al. developed an SCS approach in which various pulsed electric signals are multiplexed in space and time to target glial cells and neurons differentially. They found that differential target multiplexed programming (DTMP) provided significant improvements in both mechanical and thermal hypersensitivity in SNI rats (see Figure 2) (31). Improvement in mechanical hypersensitivity was also significantly better than that obtained using LR or HR SCS.

**Figure 3:**
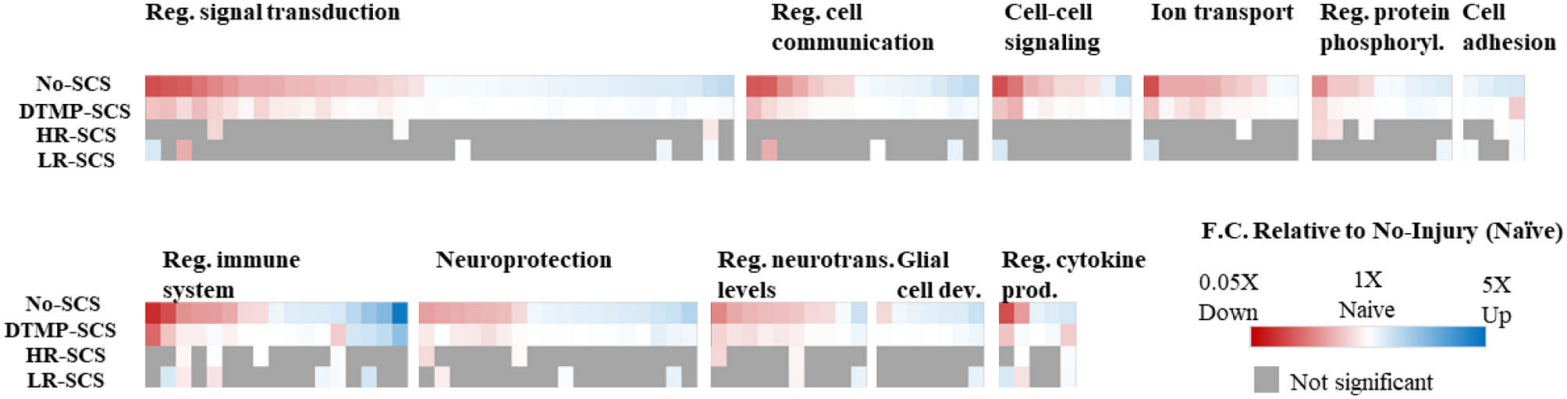
Heat maps illustrating expression levels due to the effect of DTMP, LR, and HR SCS on genes enriched in biological processes that involve neuron–glial interactions, compared with the effect of the pain model (No-SCS). The white color indicates that the expression is that of a naïve animal. Reproduced from Vallejo et al. (31). DTMP, differential target multiplexed programming; HR, high rate; LR, low rate; SCS, spinal cord stimulation.

Transcriptomics validated that, relative to naïve animals, the SNI upregulated hundreds of genes involved in regulating the immune system, inflammation, and signal transduction. DTMP modulated more of these genes than both HR and LR SCS. More importantly, DTMP significantly reversed the expression levels of 166 of such genes within 10% of the expression levels found in naïve animals. In contrast, HR SCS and LR SCS only modulated 70 and 91 of such genes, respectively, within 10% of the naïve expression levels. DTMP also reduced expression levels of genes associated with microglia and astrocyte activation (i.e., *Itgam* and *Gfap*, respectively), which the pain model had increased. This work also illustrated that DTMP provides a more substantial modulatory effect on genes associated with pain-related processes than HR and LR SCS (see Figure 3). These results indicate that DTMP may provide its analgesic effect through modulation of immune-related processes, synaptic signaling, and neurotransmission and rebalancing neuronal-glial interactions that the onset of neuropathic pain had perturbed.

**Figure 4:**
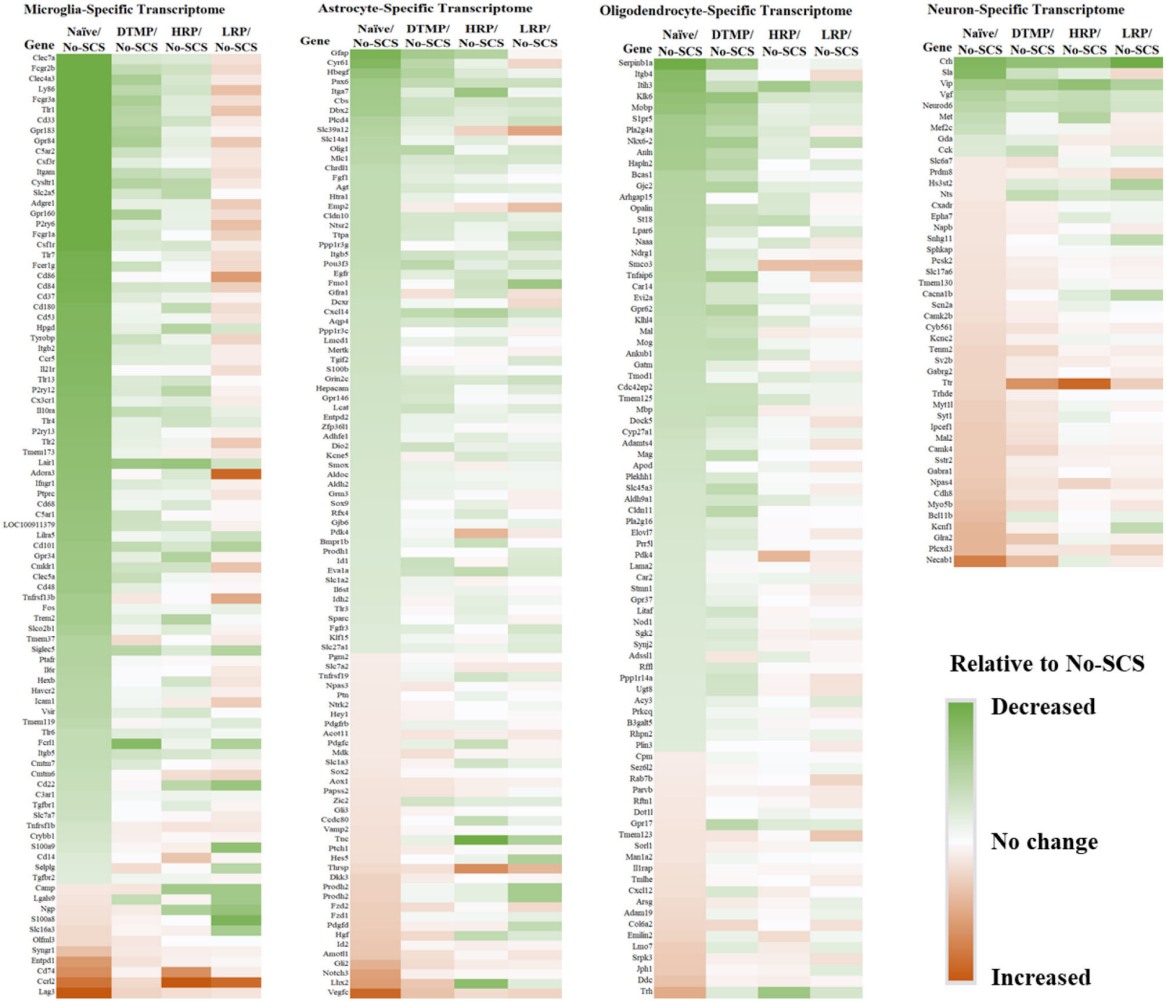
Heat maps for cell-specific transcriptomes illustrating differential expression levels (i.e., fold changes) of genes in naïve, DTMP-treated, HR-treated, and LR-treated animals relative to untreated (No-SCS) animals. From left to right: microglia-specific, astrocyte-specific, oligodendrocyte-specific, neuron-specific transcriptomes. Reproduced from Cedeno et al. (32). DTMP, differential target multiplexed programming; HR, high rate; LR, low rate; SCS, spinal cord stimulation.

In an analysis of cell-specific transcriptomes, Cedeño et al. (32) demonstrated that DTMP is more effective at modulating neurons and glial cells (microglia, astrocytes, and oligodendrocytes) transcriptomes toward the gene profile found in naïve animals (Figure 4). For instance, neuron (72 genes), microglia (101 genes), astrocyte (188 genes), and oligodendrocyte (154 genes) transcriptomes of DTMP-treated animals significantly correlated positively and strongly with that of naïve animals (R_Pearson_ ≥ 0.65). In contrast, for HR-treated animals, only the microglia transcriptome significantly correlated positively and strongly (R_Pearson_ = 0.61) with the naïve one, while the neuron transcriptome significantly correlated positively and moderately (R_Pearson_ = 0.41). Furthermore, no cell-specific transcriptome of LR-treated animals correlated strongly. Instead, the microglia transcriptome of LR-treated animals significantly correlated negatively and weakly (R_Pearson_ = −0.20) with the naïve profile, meaning that many microglia genes were further upregulated by LR SCS relative to the SNI effect.

**Figure 5:**
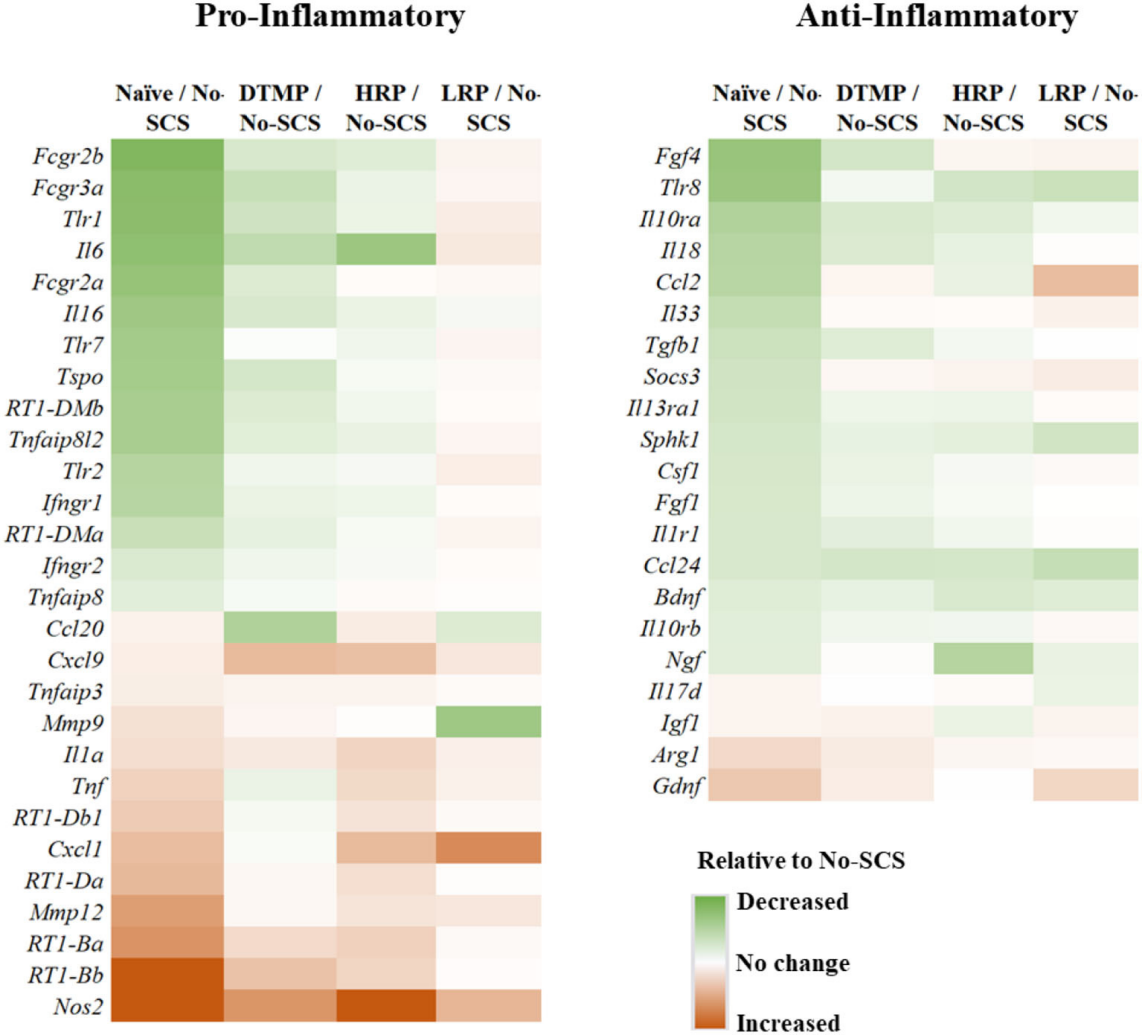
Heat maps illustrating differential expression levels of microglia-related genes known to be involved in pro- and anti-inflammatory processes for naïve, DTMP-treated, HR-treated, and LR-treated animals relative to untreated animals (No-SCS). Reproduced from Smith et al. (33). DTMP, differential target multiplexed programming; HR, high rate; LR, low rate; SCS, spinal cord stimulation.

This result is congruent with the previous findings on the effects of LR SCS on microglia activation. In further work, Smith et al. (33) reported on the effects of DTMP, HR, and LR on microglia transcriptomes associated with their resting state and states associated with pro-inflammatory processes (so-called M1-like) and neuroprotective processes (so-called M2-like). In agreement with Cedeño et al., it was found that DTMP provided the largest modulatory effect on the SNI. The microglial transcriptomes of DTMP-treated animals significantly correlated positively and strongly (R_Pearson_ = 0.58–0.65) with naïve profiles. HR treatment also produced significant positive correlations although moderate (R_Pearson_ = 0.42–0.48). Although LR treatment also produced significant positive correlations, only the resting microglia transcriptome correlated moderately with the naïve profile (R_Pearson_ = 0.39). Both the M1-like and M2-like transcriptomes correlated weakly (R_Pearson_ = 0.17). A further look at selected microglia genes within these transcriptomes, which had been reported in the literature to be associated with pro- and anti-inflammatory processes, clearly indicated that treatments with DTMP and HR better match (Figure 5) the profile of naïve animals. On the other hand, LR treatment further upregulated expression levels of pro-inflammatory genes that the SNI had increased.

Another important result of Cedeño et al. (32) is that DTMP provided strong modulation of astrocyte-specific and oligodendrocyte-specific genes, with more than 65% of genes modulated back to within 15% of their naïve levels (with more than 78% expression recovery). These glial cell types are the most abundant in the spinal cord (57), constituting about 80% of the combined microglia, astrocyte, and oligodendrocyte populations. The role of astrocytes in chronic neuropathic pain is well-established (19). Thus, a reversal of expression levels by DTMP toward naïve levels indicates a rebalancing of neuron-astrocyte interactions at synapses. An understanding of the role of oligodendrocytes in neuropathic pain is emerging. Ablation of oligodendrocytes in murine spinal cords induced neuropathic pain-like behavior (20). For instance, SNI increased expression levels of *Mobp*, an oligodendrocyte marker. An increase in expression levels of the protein encoded by this gene (myelin oligodendrocyte basic protein) was found in patients with neuropathic pain associated with HIV infection (21). Expression levels of the gene *S1pr5*, which is only expressed by oligodendrocytes, were also increased by the SNI. This gene is associated with signaling via sphingosine-1-phosphate (S1P) that triggers the migration of OPCs. Both of these genes were significantly modulated by DTMP toward naïve expression levels (31).

### Modulation of Glia-Related Protein Expression by SCS

**Figure 6:**
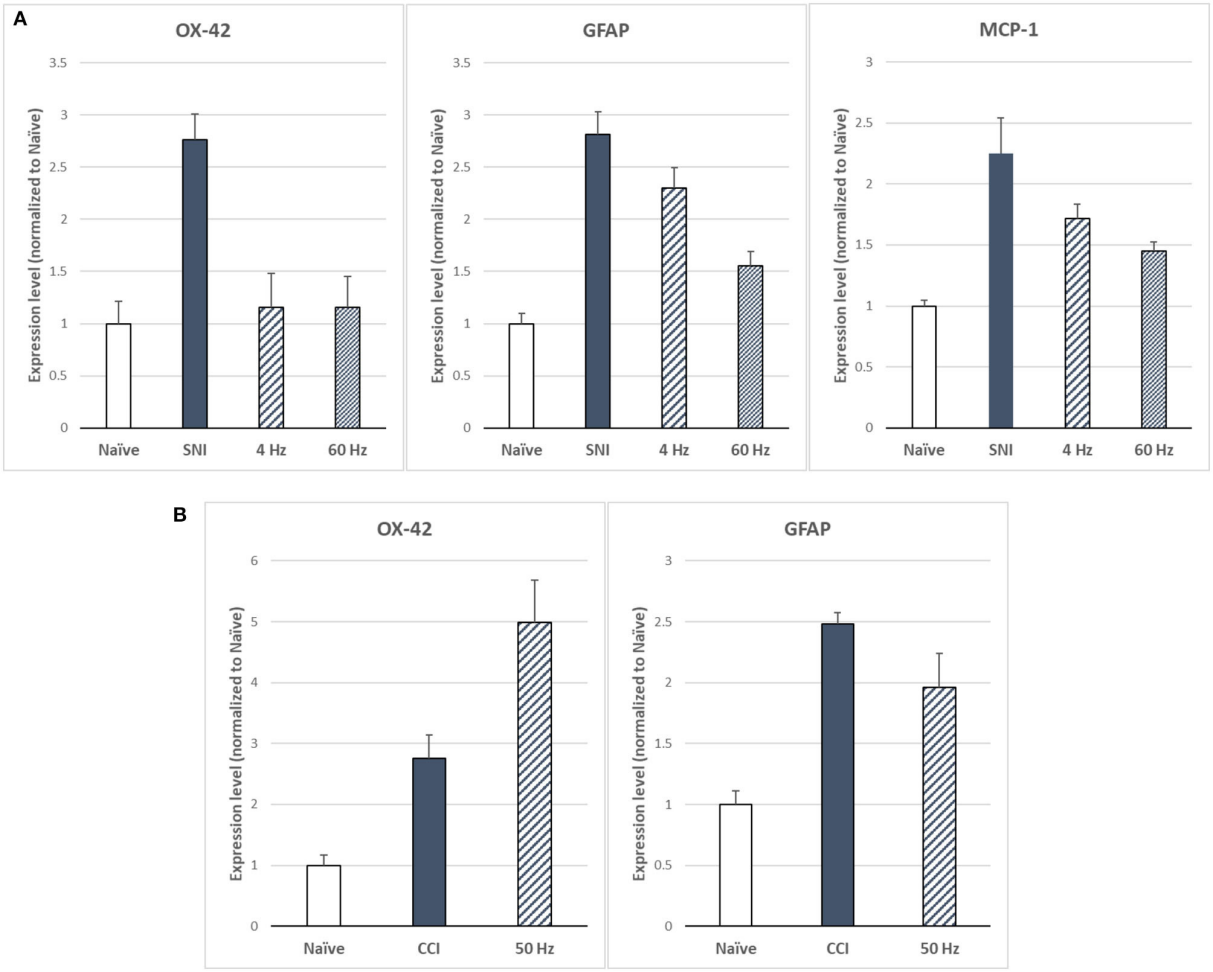
**(A)** Expression levels of microglia marker OX-42 and astrocyte markers GFAP and MCP-1 as based on Sato et al. (27) for LR SCS at 4 or 60 Hz (250 μs PW, 90% MT, 6 h/day for 4 days) in the SNI model (14 days). **(B)** Expression levels of microglia marker OX-42 and astrocyte marker GFAP based on Shu et al. (38) for LR SCS at 50 Hz [200 μs PW, 80% MT, 3h (2 × day) for 3.5 days] in the CCI model (18 days). SCS, spinal cord stimulation.

**Figure 7:**
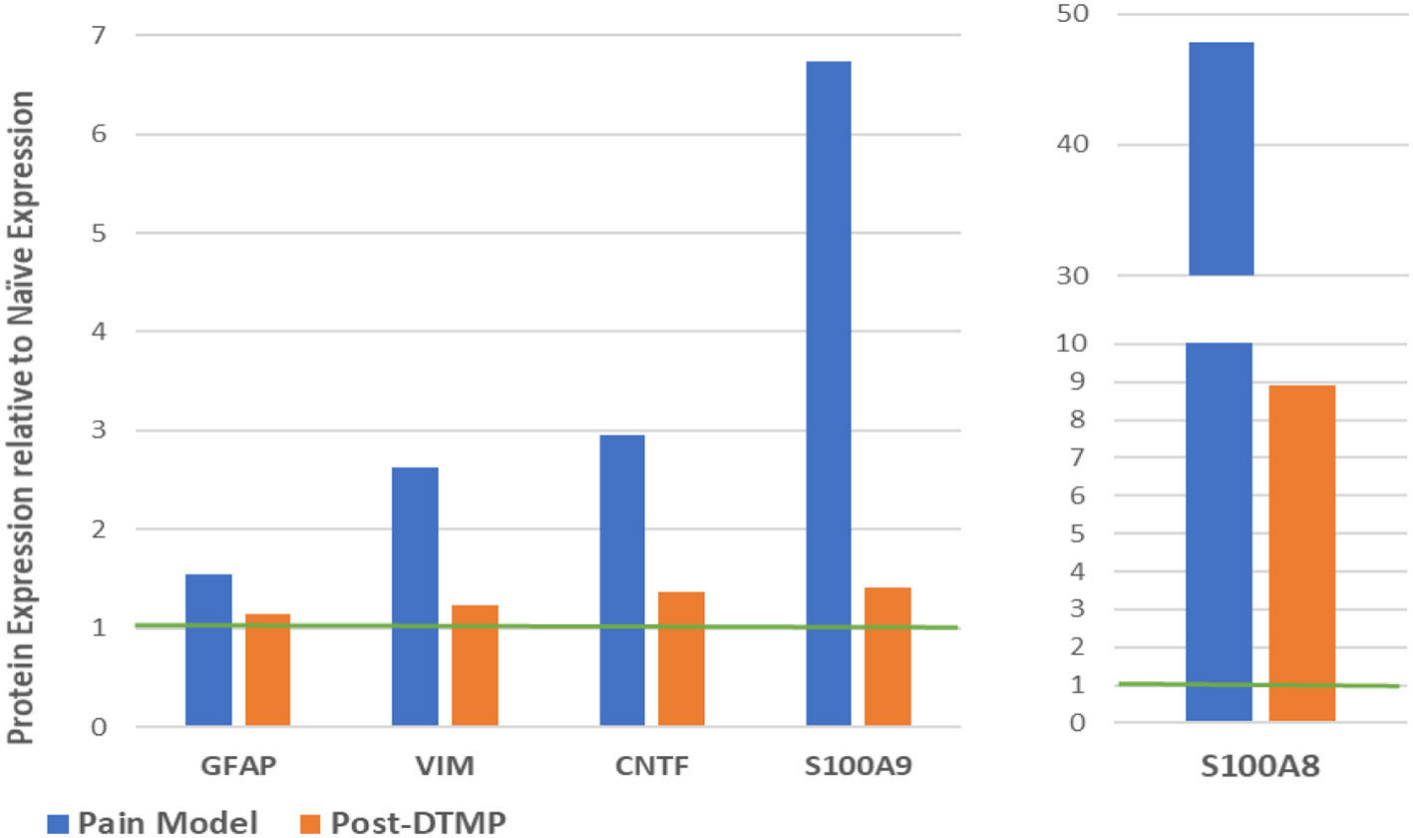
Modulatory effect of DTMP on protein expression levels of astrocyte-related proteins in the spinal cords of SNI (pain model). The green line denotes the normalized expression level in naïve animals. DTMP, differential target multiplexed programming; SNI, spared nerve injury.

Sluka et al. (27) reported first on the effect of SCS on glia-related protein expression in the dorsal horn of SNI animals. They found that the expression levels of OX-42 (also known as ITGAM, a marker of microglial activation), MCP-1 (also known as CCL2), and GFAP (markers of astrocyte activation) were significantly elevated, relative to naïve animals, after 14 days of the SNI (Figure 6A). They found that LR SCS treatment for 6 h/day for 4 consecutive days at either 4 or 60 Hz significantly decreased expression levels of these markers. Interestingly, in a recent study, Shu et al. (38) found that although expression levels of GFAP and OX-42 were significantly elevated by the CCI (18 days) relative to naïve animals (Figure 6B), LR SCS treatment at 50 Hz (6 h/day in two 3 h sessions for 3.5 days) did not decrease the expression of these proteins. In contrast, they found that LR SCS significantly increased the expression of OX-42 relative to the expression in untreated animals. Lack of congruence on the effects of LR SCS with the previous work by Sato et al. was attributed, in part, to experimental differences (animal models, SCS protocols, and post-injury times). Preliminary results of a proteomics-based analysis in our laboratory show that continuous 48 h of DTMP significantly decreased expression levels of astrocyte markers GFAP and VIM, which had been significantly increased by the SNI (Figure 7). Besides these two proteins, the SNI also upregulated S100A8 and S100A9, calcium-binding proteins known to induce astrocyte differentiation in inflammatory processes, and CNTF, a protein expressed by astrocytes during gliosis. DTMP decreased their expression levels toward naïve levels. The study also found that the pain model increased expression levels of three phosphorylated isoforms of GFAP (p-GFAP at residues T31, S80, and T148) and 11 phosphorylated isoforms of VIM (p-VIM at residues S7, S10, S18, S39, S51, S56, S73, S325, S430, T436, and S549). DTMP decreased expression levels of the three p-GFAP isoforms and 10 of the p-VIM isoforms toward levels in naïve animals. Although the role of these phosphorylated isoforms has not been elucidated, phosphorylation and dephosphorylation of filament proteins such as GFAP and VIM may be associated with signaling processes in which the phosphorylated isoforms are intermediate states linked with processes tightly associated with it such as neurotransmission regulation (i.e., glutamate or GABA buffering) and calcium-mediating signaling of inflammatory pathways.

Astrocytes play an important role at the synaptic cleft, where they monitor the homeostatic balance of nutrients, ions, and neurotransmitters. An analysis of the proteomics of the effect of DTMP on the regulation of ion transport within the spinal cord of SNI animals found that proteins expressed by astrocytes are key elements in the establishment of neuropathic pain and the analgesic effect of DTMP. For example, KIR4.1 is a potassium ion (K^+^) channel that allows entry of K^+^ into astrocytes while inhibiting the release of BDNF mediated by astrocytic Na^+^/K^+^ ATPases such as ATP1A2 and ATP1B2. KIR4.1, ATP1A2, and ATP1B2 were found to be upregulated by DTMP. Buffering of K^+^ into the astrocytes facilitates the activity of neuronal KCC2, a K^+^/Cl^−^ symporter that maintains the homeostatic balance of chloride in neurons, which is known to play an important role in GABA-regulated post-synaptic inhibition. DTMP also reversed the effect of the SNI on the enzymes PHGDH and PSAT1, involved in the synthesis of L-serine, an essential amino acid in the production of neurotransmitters D-serine and glycine. Neurons cannot synthesize L-serine. The reduction of PHGDH levels has been previously associated with the reduction of L-serine and neuropathic pain. Thus, an increase of PHGDH and PSAT1 by DTMP treatment is congruent with the important role of glial synthesis of important nutrients that keep homeostatic balance in the synapsis. Further emphasis is on the modulating role of DTMP on astrocytes in regulating ionotropic and metabotropic glutamate (GLU) receptors. For instance, the SNI significantly decrease expression levels of the metabotropic glutamate receptor MGLUR5, which DTMP reversed. Neuropathic pain also affects intracellular second messenger pathways that involve calcium ions (Ca^2+^). Large concentrations of intracellular Ca^2+^ in glial cells are associated with inflammatory pathways. The SNI increased the expression of IP3R1, a protein of the endoplasmic reticulum that aids the release of Ca^2+^ into the cytoplasm. DTMP significantly reversed its expression. The decrease of intracellular Ca^2+^ in astrocytes would reduce its release into gap junctions that link astrocytes to each other in propagating calcium waves, which is considered a key process in the sensitization of distal neural tissues. Further analyses of the role of calcium signaling in the SNI and the effects of DTMP are underway. The investigations into the modulatory role of DTMP on the NFkB signaling pathway in inflammation and neuroprotective role *via* modulation of the caspase-apoptosis pathway.

## Concluding Remarks and the Next Frontiers

Recognizing the role of glial cells in chronic neuropathic pain is a relatively recent advancement in our understanding of its mechanism of action, particularly in a line of thought that focused on a doctrine that has placed neurons as the only active player. If this fact is accepted, it is also rational to think that treatments for chronic neuropathic pain must also account for their presence and role. Until recently, the field of SCS was bound to a theory of pain that, although useful for its development and advancements, had ignored many fundamental processes related to the interactions of the neurons with their surrounding glial cells. The advent of molecular biology tools has also provided an opportunity to explore neural tissues beyond what electrophysiological measurements could tell us from the perspective of neuronal behavior. These tools have opened the door to a molecular understanding of biological processes associated with pain that can facilitate the optimization of SCS approaches, targeting both neurons and glial cells in such a way that their interactions, perturbed by the onset and persistence of chronic pain, can be rebalanced. The recent preclinical work highlighted in this report should encourage others to move into the next frontiers in our understanding of the mechanism of action of SCS and the value of this comprehension to improving clinical outcomes. At the preclinical level, we need to understand further the effects of the electrical signals applied in SCS at the cellular level and the development of chronic pain. Cell sorting techniques and single-cell RNA sequencing would provide more specificity. *In situ* proteomics and peptidomics of neural tissues, using laser-based desorption ionization techniques (such as MALDI) coupled with tandem MS/MS will also provide a way of “imaging” the spatial distribution of proteins in neural tissue and how pain models and treatments affect these. The investigation of other post-translational changes in proteins (acetylation, glycosylation, etc.) would also help understand the persistence of chronic pain since particular isoforms of modified proteins may drive this. Other interesting frontiers include the link between epigenetics and chronic pain, the potential role of genetic predisposition, and environmental factors in establishing and maintaining chronic pain. In a more practical sense, clinical validation of the hypotheses formulated by preclinical discoveries is perhaps the next frontier. This would require searching for suitable pain-related biomarkers that can be identified and quantitated in easily accessible fluid samples or using imaging techniques such as MRI.

## Author Contributions

DC and RV designed the outline of the manuscript. DC produced the first draft. All authors contributed equally to the final version of the manuscript.

## Conflict of Interest

DC and RV are advisory board members and paid consultants of Medtronic, Inc., co-inventors of patents related to differential target multiplexed SCS. KC is a consultant to Medtronic Inc. DC and RV are cofounders of Lumbrera LLC. The remaining author declares that the research was conducted without any commercial or financial relationships that could be construed as a potential conflict of interest.
